# The Great Imitator: A Case of Secondary Neurosyphilis With Unusual Neurological Manifestations

**DOI:** 10.7759/cureus.96547

**Published:** 2025-11-11

**Authors:** Naveeda Mahar, Saravanaa Sankar, Vinod Warrier, Shahid Nasim, Alekya Siddabathula

**Affiliations:** 1 Acute Medicine, Mid and South Essex NHS Foundation Trust, Southend-on-Sea, GBR; 2 Internal Medicine, Southend University Hospital NHS Foundation Trust, Southend-on-Sea, GBR; 3 Acute Medicine, Southend University Hospital NHS Foundation Trust, Southend-on-Sea, GBR; 4 Internal Medicine, Mid and South Essex NHS Foundation Trust, Southend-on-Sea, GBR; 5 Emergency Medicine, Mercy General Hospital, St. Louis, USA

**Keywords:** atypical presentation, bell's palsy mimic, cn vii palsy, diagnostic challenge, maculopapular rash, peripheral neuropathy, secondary neurosyphilis, sti

## Abstract

Neurosyphilis, a central nervous system infection caused by the bacterium *Treponema pallidum*, is becoming increasingly rare due to the widespread use of antibiotics. However, unusual presentations continue to pose diagnostic challenges. We report a case where a patient presented with progressive neurological symptoms, initially mimicking other systemic and neurological disorders.

A 52-year-old man presented with abdominal pain associated with fever and loose stools with per rectal bleeding and accompanied by progressive lower limb paresthesia and numbness. Over subsequent visits, he developed a widespread maculopapular rash, right-sided Bell’s palsy, and ascending sensory loss involving the perianal and genital regions. Serial laboratory investigations initially revealed elevated inflammatory markers and abnormal liver function tests, which gradually improved. Initial viral and autoimmune screens were inconclusive. The MRI of the brain and spine was unremarkable; however, the CSF analysis revealed mildly elevated protein levels. On further testing, *Treponema pallidum* serology returned positive, confirming secondary neurosyphilis. He received intravenous ceftriaxone 2 g once daily for 10 days, leading to complete resolution of symptoms.

This case highlights variable features of secondary syphilis, which can resemble a range of systemic, infectious, and neurological conditions. Furthermore, initially, the patient denied any history of unprotected intercourse. Following the positive syphilis serology results, he acknowledged recent, unprotected sexual activity while on holiday. Therefore, a key learning point is that clinicians need to create a confidential and supportive environment that facilitates accurate disclosure, thereby aiding in timely diagnosis and treatment.

Secondary neurosyphilis can present with various neurological and systemic features, often disguised as other disorders. Early detection and antibiotic therapy are vital for complete recovery. This case also highlights the importance of gathering confidential and non-judgmental information to uncover essential details that directly impact patient management and outcomes.

## Introduction

Syphilis is a sexually transmitted infection caused by the spirochete *Treponema pallidum*. The clinical manifestation depends on the stage of the disease. Although once highly prevalent during the pre-antibiotic era, following the introduction of penicillin in the mid-20th century, the global incidence of syphilis declined significantly. However, in recent years, an increase in incidence has been noticed in both developed and developing countries, often attributed to the changing sexual behaviors, reduced awareness, and gaps in screening and early diagnosis [1,2]. According to the World Health Organization, an estimated 8.0 million new infections occurred in adults aged 15-49 years in 2022, compared to 7.1 million in 2020, with a global prevalence of approximately 0.6% [3]. Data from the Global Burden of Disease Study also demonstrated an increase in the annual incidence of cases, from 8.9 million in 1990 to 14.1 million in 2019 [4]. These trends highlight the ongoing public health challenge of syphilis and the importance of clinical vigilance, comprehensive sexual health education, and accessible diagnostic and treatment services to curb further transmission.

Neurosyphilis refers to infection of the central nervous system by *Treponema pallidum*, which can occur at any time after initial infection. Classically, neurosyphilis is categorized into early (meningitis, meningovascular forms, ischemia/infarct), late (general paresis and tabes dorsalis), and atypical neurosyphilis. Secondary neurosyphilis, although rare, can present with a combination of dermatological, gastrointestinal, neurological, musculoskeletal, and systemic symptoms, making its diagnosis particularly challenging [5]. Early recognition and treatment are crucial in preventing complications.

This report follows the diagnostic journey of a patient with evolving systemic and neurological features, whose initial low-risk profile delayed consideration of syphilis. This case highlights the importance of maintaining a broad differential diagnosis of unexplained neurological symptoms and how revisiting a confidential sexual history can be pivotal to reaching a correct diagnosis earlier.

## Case presentation

A 52-year-old male presented to the emergency department with a four-day history of abdominal discomfort, fever, night sweats, and loose stools streaked with blood. He also reported pain during defecation and tingling sensations in both feet that had been ascending upwards over the past few days. Over the preceding months, he had experienced unintentional weight loss, reduced appetite, and increased fatigue. The patient was a non-smoker, consumed 14 units of alcohol weekly, and denied any history of unprotected sexual activity.

Upon examination, he appeared systemically unwell but hemodynamically stable. Mild epigastric tenderness was noted, with no other abnormalities on physical examination. Baseline investigations showed elevated CRP, thrombocytosis, and deranged liver function tests (LFTs) (Table 1). A CT of the abdomen was unremarkable. Given the clinical presentation, viral hepatitis or drug-induced hepatitis was suspected, and the patient was managed supportively.

**Table 1:** Comparison of blood test results: initial vs. follow-up weeks later CRP: C-reactive protein, ALT: alanine aminotransferase, AST: aspartate aminotransferase, GGT: gamma-glutamyl transferase, HCT: hematocrit, RCC: red cell count, RDW: red cell distribution width, MCV: mean corpuscular volume, MCH: mean corpuscular hemoglobin

Blood tests				
Test	Initial result	Repeat result (6 weeks)	Unit	Normal range
CRP	11	3	mg/L	<5
Full blood count				
Hemoglobin	136	132	g/L	130-180
White cell count	6.4	5.6	10⁹/L	4.0-11.0
Platelet count	466	302	10⁹/L	150-400
HCT	0.40	0.39	L/L	0.40-0.52
RCC	4.50	4.46	10¹²/L	4.5-6.5
RDW	13.4	13.4	%	11.0-14.8
MCV	89.3	87.8	fL	80-100
MCH	30.1	29.6	pg	27.0-32.0
Differential count				
Neutrophil count	3.51	3.60	10⁹/L	1.7-7.5
Lymphocyte count	2.23	1.24	10⁹/L	1.0-4.5
Monocyte count	0.57	0.59	10⁹/L	0.2-0.8
Eosinophils	0.06	0.13	10⁹/L	0.0-0.4
Basophils	0.03	0.03	10⁹/L	0.0-0.1
Liver function tests				
Total protein	80	69	g/L	60-80
Albumin	41	39	g/L	35-50
Globulin	37	30	g/L	20-35
Alkaline phosphatase	577	144	U/L	30-130
Total bilirubin	19	6	umol/L	0-21
ALT	241	56	U/L	<50
AST	357	–	U/L	<50
GGT	1122	–	U/L	<55
Urea and electrolytes				
Sodium	137	138	mmol/L	133-146
Potassium	4.8	4.0	mmol/L	3.5-5.3
Urea	6.1	7.0	mmol/L	2.5-7.8
Creatinine	77	75	umol/L	59-117

Three weeks later, he returned with new symptoms. A non-itchy, non-tender maculopapular rash had appeared on his torso, gradually spreading to his arms. The tingling or numbness in his feet had now progressed up to mid-thigh. With numbness reported in the genital and anal areas as well. He also reported having difficulty passing urine and stool. A neurological examination confirmed bilateral peripheral neuropathy in the lower limbs, with a normal PR examination. His blood tests showed improved LFTs with a negative viral screen, but neurological signs and symptoms prompted consideration of a broader differential and further investigations as an outpatient.

Shortly thereafter, the clinical story took a striking turn. He attended A&E the very next day with an acute onset of right-sided facial droop and numbness, initially raising suspicion of stroke. He also reported having worsening perianal paresthesia and was not able to feel when passing urine or stool, with the rash now extending to his lower limbs. Examination revealed a lower motor neuron pattern of facial palsy with worsening neuropathy. As the CT head was unremarkable, he was commenced on steroids with PPI cover for presumed Bell's palsy. He was admitted for further workup to investigate a possible subacute myelo polyneuropathic condition, demyelinating disorder, or mononeuritis multiplex.

**Figure 1:**
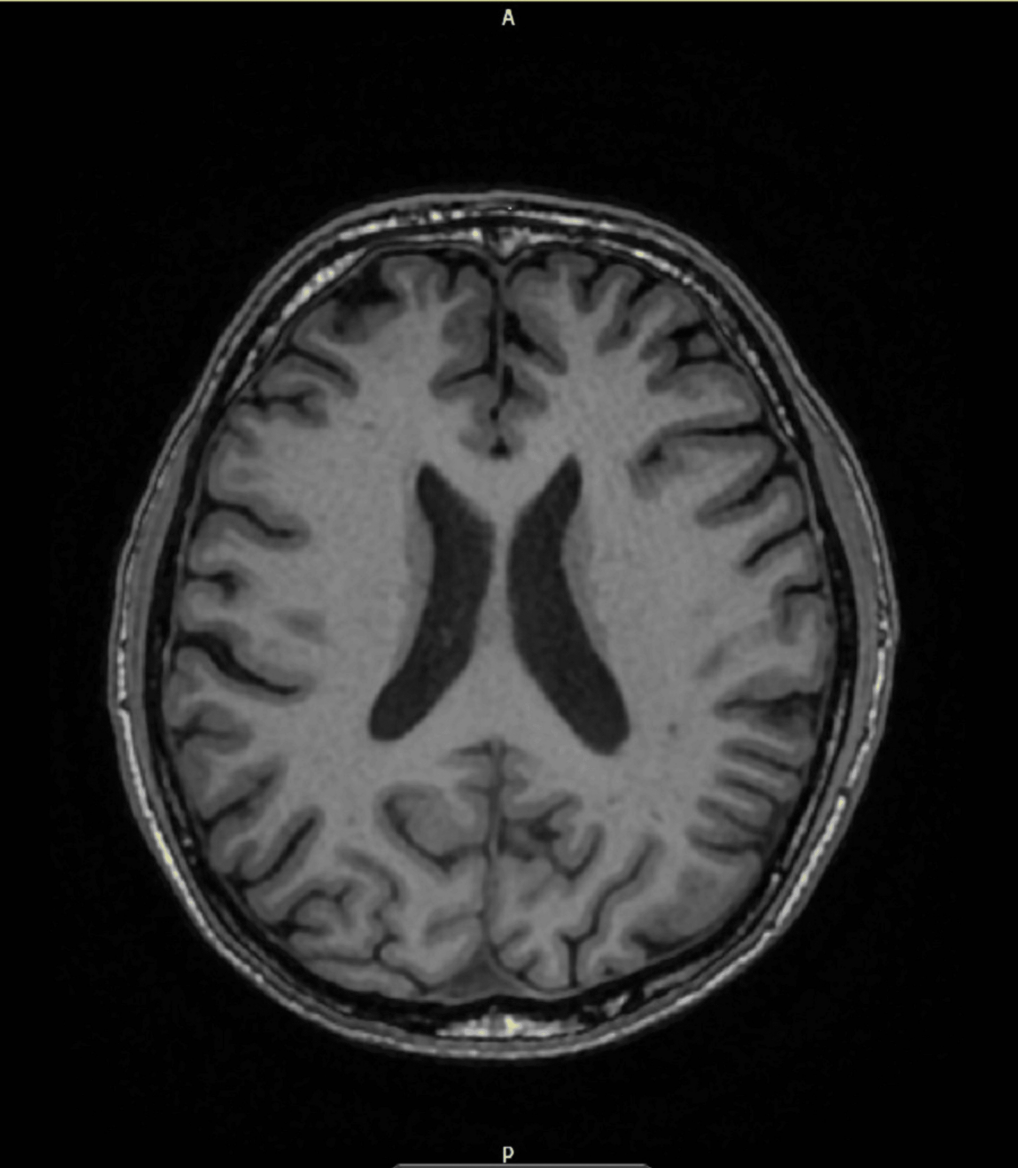
Normal MRI brain There’s no sign of intracranial pathology or demyelinating disorder with normal CSF appearance. Therefore, the MRI is basically unremarkable. Excluding potential differentials. CSF: cerebrospinal fluid, MRI: magnetic resonance imaging

**Figure 2:**
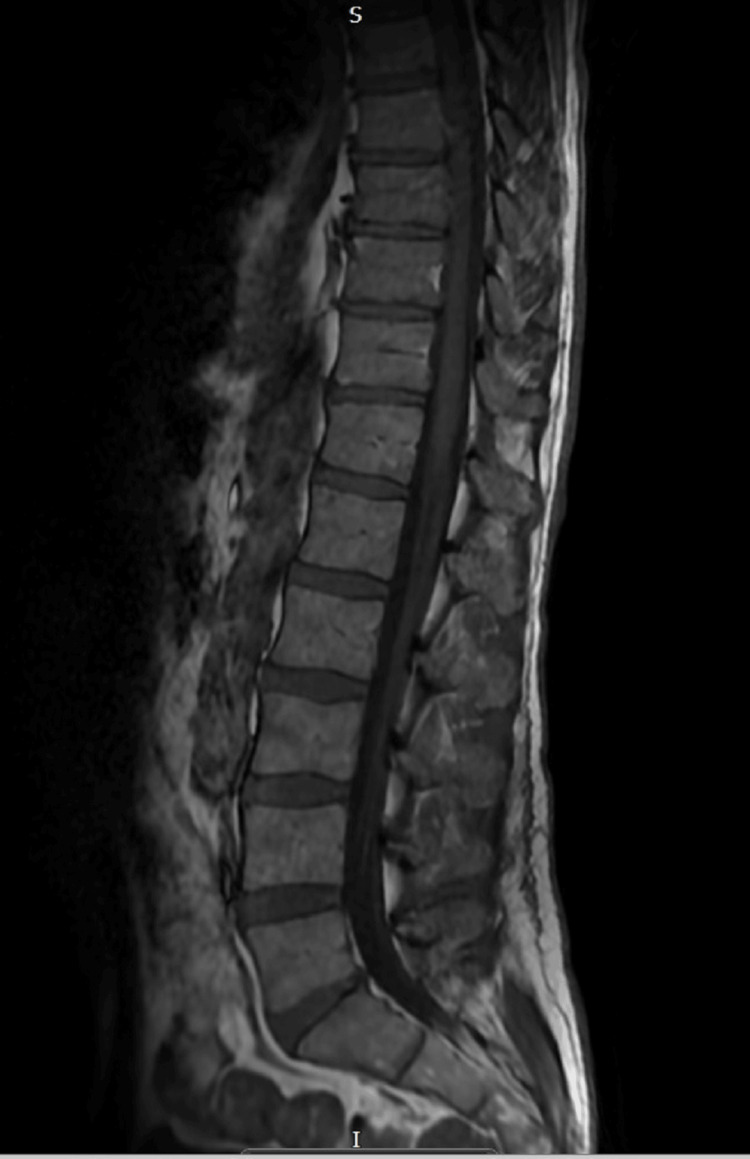
Normal MRI spine Unremarkable appearances of the cord, conus, and cauda equina. MRI: magnetic resonance imaging

During his stay in the hospital, his sensory deficit deepened, and he now complained of loss of temperature sensation in the upper part of both thighs, along with the persistent paraesthesia. On examination, persistent CN VII palsy was noted with persistent peripheral neuropathy and saddle paresthesia, but no loss of anal tone. MRI of the brain and whole spine revealed mildly enlarged bilateral lymphadenopathy, likely reactive, and a lumbar puncture demonstrated mildly elevated proteins in the CSF (Figures 1-2, Table 2). Given the patchy neurological findings, the spectrum of autoimmune and infectious screens was broadened. He was discharged home with a plan to gradually taper the steroids and follow up in the clinic after the nerve conduction study as an outpatient.

During the next follow-up review a week later, his Bell’s palsy signs had improved. However, he still had a gradually worsening rash with new right knee arthralgia with effusion. By this time, syphilis serology requested while he was admitted had returned positive, revealing the true culprit (Table 3) [6]. On further discussion conducted in confidential settings, the patient acknowledged a history of unprotected sexual contact during a recent holiday, which wasn’t disclosed earlier. Hence, a diagnosis of neurosyphilis was made, further supported by confirmatory CSF results, and he was started on ceftriaxone 2 g once daily for 10 days due to the unavailability of benzathine penicillin, in accordance with the national guidelines (Table 2) [7-9].

**Table 2:** CSF analysis A lumbar puncture was repeated after the syphilis serology came back positive to confirm the diagnosis of neurosyphilis. CSF: cerebrospinal fluid, WBC: white blood cell(s), RBC: red blood cell(s), VDRL: venereal disease research laboratory, TPPA: *Treponema pallidum* particle agglutination

CSF analysis			
Test	Result	Units	Reference range
CSF glucose	4.5	mmol/L	≈ ⅔
CSF total protein	0.66	g/L	0.15–0.45 g/L
CSF appearance	Clear	-	-
Oligoclonal banding	Negative (serum negative)	-	-
Culture report	No growth	-	-
WBC differential			
Polymorphs	0	%	-
Lymphocytes	100	%	-
Cells			
White blood cells	12	cells/mm³	-
Red blood cells	50	cells/mm³	-
Gram stain	No organisms seen	-	-
Neurosyphilis			
CSF VDRL	Positive	-	-
CSF TPPA	Positive	-	-

**Table 3:** Syphilis serology EIA: enzyme immunoassay, IgM: immunoglobulin M, ELISA: enzyme-linked immunosorbent assay, TPPA: *Treponema pallidum* particle agglutination, RPR: rapid plasma reagin

Test	Results
Syphilis (treponemal) antibody	Detected
Syphilis total antibody EIA	Positive
Syphilis IgM ELISA	Positive
TPPA	Positive
RPR	Positive 1:32

Follow-up after completion of the antibiotic course showed gradual but complete resolution of his symptoms. He remained well, with no recurrence or new symptoms, at subsequent follow-up visits, and had negative serology and normal CSF results at six months.

## Discussion

Neurosyphilis is often described as a "great imitator" that remains strikingly relevant in modern clinical practice. Its ability to mimic can lead even experienced clinicians down the alternate diagnostic pathways [10-11]. This case encapsulates that challenge: a patient who initially presented with constitutional and gastrointestinal symptoms, whose evolving neurological features gradually unraveled a diagnosis that was not immediately suspected.

The early features (abdominal pain, loose stools, and fever with deranged LFTs) naturally directed clinicians toward more common diagnoses such as hepatitis; however, subsequent viral and autoimmune screens came back negative. The neurological signs evolved more gradually, initially presenting as an ascending peripheral neuropathy, followed by cranial nerve palsy and a widespread but patchy neuropathy, closely resembling mononeuritis multiplex, some inflammatory neuropathies, or even subacute myelopathies [12]. Each stage appeared clinically coherent in isolation, but it was the longitudinal pattern that held the key to this case.

While cranial nerve involvement in neurosyphilis is well documented, facial nerve palsy as an initial neurological manifestation remains uncommon [13-14]. This highlights the importance of considering a broader differential diagnosis for atypical or progressive cranial neuropathies, particularly when associated with other neurological, dermatological, or systemic symptoms.

From a diagnostic point of view, the CSF showing mildly elevated proteins, with the MRI being essentially unremarkable, could have been falsely reassuring. However, neurosyphilis, particularly in early or secondary forms, may present with subtle or non-specific findings on CSF analysis and imaging [15]. Therefore, serological testing should be considered early on, especially in unexplained peripheral or cranial neuropathies.

A crucial step in this case was not imaging or laboratory data but revisiting the patient’s history in a confidential and non-judgmental manner. Initially, the patient denied unprotected sexual activity, which lowered clinical suspicion. But later, before disclosing the results, further discussion revealed relevant exposure. This emphasizes thorough, confidential, and sometimes repeated sexual history taking, as the patient may withhold information.

The uniqueness of this case lies in its unusual sequence of manifestations, beginning with gastrointestinal symptoms and evolving into multifocal neuropathy with facial palsy. This uncommon presentation highlights the continued need for clinician awareness of syphilis in differential diagnoses.

## Conclusions

This case highlights the importance of considering neurosyphilis in the differential diagnosis of patients with unexplained neurological symptoms, even in those with a seemingly low risk profile. Like in our patient who initially presented with hepatitis, but then worsening peripheral neuropathy with a rash progressing to facial nerve palsy prompted us to broaden the spectrum of investigations. A normal MRI of the brain and spine, with negative autoimmune, viral, and vasculitic screens (excluding other differentials), and persistent symptoms, as well as increased proteins and lymphocytes on CSF analysis, guided us to consider an infectious etiology. Eventually leading to a delayed diagnosis of syphilis. Atypical presentations, such as facial nerve involvement or isolated peripheral neuropathy, may delay the diagnosis. Therefore, early consideration of serological testing, thorough history-taking, and a broad differential diagnosis are crucial for the timely identification and treatment of neurosyphilis, preventing long-term complications.
